# Metabolic syndrome for the prognosis of postoperative complications after open pancreatic surgery in Chinese adult: a propensity score matching study

**DOI:** 10.1038/s41598-023-31112-x

**Published:** 2023-03-08

**Authors:** Yuanqiang Dai, Yaping Shi, Heng Wang, Tianhua Cheng, Boyang Xia, Yu Deng, Tao Xu

**Affiliations:** Faculty of Anesthesiology, Changhai Hospital, Naval Medical University, No.168 Changhai Road, Shanghai, 200433 China

**Keywords:** Nephrology, Diseases, Pancreatic disease, Gastroenterology

## Abstract

To investigate the relationship between metabolic syndrome (MS) and postoperative complications in Chinese adults after open pancreatic surgery. Relevant data were retrieved from the Medical system database of Changhai hospital (MDCH). All patients who underwent pancreatectomy from January 2017 to May 2019 were included, and relevant data were collected and analyzed. A propensity score matching (PSM) and a multivariate generalized estimating equation were used to investigate the association between MS and composite compositions during hospitalization. Cox regression model was employed for survival analysis. 1481 patients were finally eligible for this analysis. According to diagnostic criteria of Chinese MS, 235 patients were defined as MS, and the other 1246 patients were controls. After PSM, no association was found between MS and postoperative composite complications (OR: 0.958, 95%CI: 0.715–1.282, *P* = 0.958). But MS was associated with postoperative acute kidney injury (OR: 1.730, 95%CI: 1.050–2.849, *P* = 0.031). Postoperative AKI was associated with mortality in 30 and 90 days after surgery (*P* < 0.001). MS is not an independent risk factor correlated with postoperative composite complications after open pancreatic surgery. But MS is an independent risk factor for postoperative AKI of pancreatic surgery in Chinese population, and AKI is associated with survival after surgery.

## Introduction

According to the American Cancer Society, pancreatic cancer is predicted to remain the second leading cause of cancer deaths within the next decade^1^. Both the incidence and death rates of pancreatic cancer are increasing globally^2^. Surgery remains the only option which could offer curative potential for pancreatic patients^3–5^. However, complications after pancreatic surgery frequently reported and considerably associated with mortality or severe clinical outcome^6,7^. Identifying the potential risk factors for postoperative complications is the first challenge for the prevention.

Metabolic syndrome (MS) is a syndrome involving a variety of metabolic disorders, and a predictor of several diseases such as cardiovascular diseases, osteoarthritis, and certain cancers^8^. The association between MS and postoperative complications after pancreatic surgery are still largely unclear and currently, only a limited number of studies have analyzed this association, showing a debating result. Some studies suggested that MS^9^ and its components like obesity^10^, diabetes^11,12^ and hypertension^13^ would increase the risk of medical complications^14–16^, overall and cancer-specific mortality^17^. Additionally, only a few of them concentrated on open pancreatic cancer surgery with controversial conclusions^10,18–20^.

According to previous studies, the prevalence and clinical diagnostic criteria of MS were different in races^9,17,21,22^. Notably, the diagnostic criteria of BMI adopted in MS for Chinese is lower than that for the Whites, which might have impact on postoperative complications after pancreatic surgery. Therefore, studies only adopted universal criteria for all race for small portion of Chinese population were not exactly correct^18,19^. This study examined a large group of Chinese patients hospitalized with pancreatic cancer who had underwent open pancreatic cancer surgery to determine if MS was associated with postoperative complications.

## Methods

### Data collection and study design

The study was conducted in accordance with the ethical standard specified by national health commission of China (Act 11, 2016) and approved by the ethics committee of Changhai Hospital (CHEC2020-170). The requirement for written informed consent was waived by the ethics committee. The clinical registration number is ChiCTR2000031167 (available on http://www.chictr.org.cn/). This study retrieved the medical records of elective pancreatectomies in Changhai Hospital from January 2017 to May 2019.

For the eligible patients in this study, relevant information was retrieved: descriptive and surgical information; diagnostic information of metabolic syndrome; information of complications during hospitalization; prognosis during hospitalization. Telephone follow-ups were made after data collections.

### Diagnosis of MS

The diagnosis of metabolic syndrome is defined on recommendations of Chinese Diabetes Society^17^: (1) Overweight and (or) obesity, BMI ≥ 25 kg/m^2^; (2) Hyperglycemia, fasting blood glucose (FPG) ≥ 6.1 mmol/l (110 mg/dl) and (or) 2 h PG ≥ 7.8 mmol/l (140 mg/dl), and (or) diabetes mellitus diagnosed and treated; (3) Hypertension, systolic/diastolic blood pressure ≥ 140/90 mmHg, and (or) hypertension diagnosed and treated; (4) Dyslipidemia, fasting blood triglyceride ≥ 1.7 mmol/L (150 mg/dl), and/or fasting blood HDL-C < 0.9 mmol/L (35 mg/dl) for male and < 1.0 mmol/L (39 mg/dl) for female. Patients qualified 3 or more of the above 4 components will be diagnosed as MS.

### Primary and secondary outcomes

Our primary outcome was the correlation of composite complications (CC) with MS. A composite of postoperative complications defining as the overall occurrence of any symptoms of the following five components during hospitalization: (1) cardiovascular and cerebrovascular events (CCE), (2) non-pulmonary postoperative infection (NPPI), (3) pulmonary complications (PC), (4) complications requiring surgical intervention (CRSI), and (5) postoperative acute kidney injury (AKI). CCE included myocardial infarction, heart failure, cardiac arrest, stroke, and pulmonary embolism. NPPI were differentiated according to the location or system, such as superficial wound infection, pancreatic fistula, surgical incision, abdominal infection, urinary infection, systemic infection. PC^10^ included pulmonary infection, atelectasis, pneumothorax, hemothorax, pleural effusion and respiratory related hypoxemia. Postoperative AKI was defined as a categorical variable according to the Kidney Disease Improving Global Outcomes work group, as any increase in postoperative serum creatinine of 0.3 mg/dL or more (to convert to micromoles per liter, multiply by 88.4) or a 50% increase from preoperative baseline serum creatinine level. The Cockcroft–Gault equation was adopted for eGFR evaluation, depending on patients’ gender. Secondary outcome were correlations of components of CC with MS and prognosis of complications.

### Statistical analysis

A propensity score matching (PSM) was performed at a 1:4 fixed ratio nearest-neighbor matching to control for bias from covariates including gender, preoperative biliary stented, and the operation method. The caliper value was 0.2. The normality of data distribution was tested by Kolmogorov–Smirnov test. Continuous variables were expressed as means ± standard deviation or medians (IQR), as appropriate for the data distribution. Group differences were assessed by ANOVA test or Mann–Whitney U tests. Categorical variables were expressed as the number of cases or the percentages (%). Chi-squared or Fisher’s exact tests were adopted for assessing the differences between groups. Given that incidences varied considerably across the four components. For the primary hypothesis that MS patients have increased risk for postoperative complications, a multivariate distinct effect generalized estimating equations model with an unstructured working correlation was employed and the odds ratios (ORs) across the five components were assessed. Binary logistic regression was selected to fit the regression model. Results were reported as a covariate-adjusted OR and its 95% confidence interval (CI) that summarizing the relationship between MS and postoperative composite complications at the 0.05 significance level. Cox regression model was used to analyze the survival of patients within 30 and 90 days after operation. A two-tailed *P* value < 0.05 was considered to be significant. All analyses were performed using IBM SPSS Statistics v21 (IBM Corporation, NY, USA) or RStudio (version 4.1.3).

## Results

### Characteristic data and outcomes in hospital

**Figure 1:**
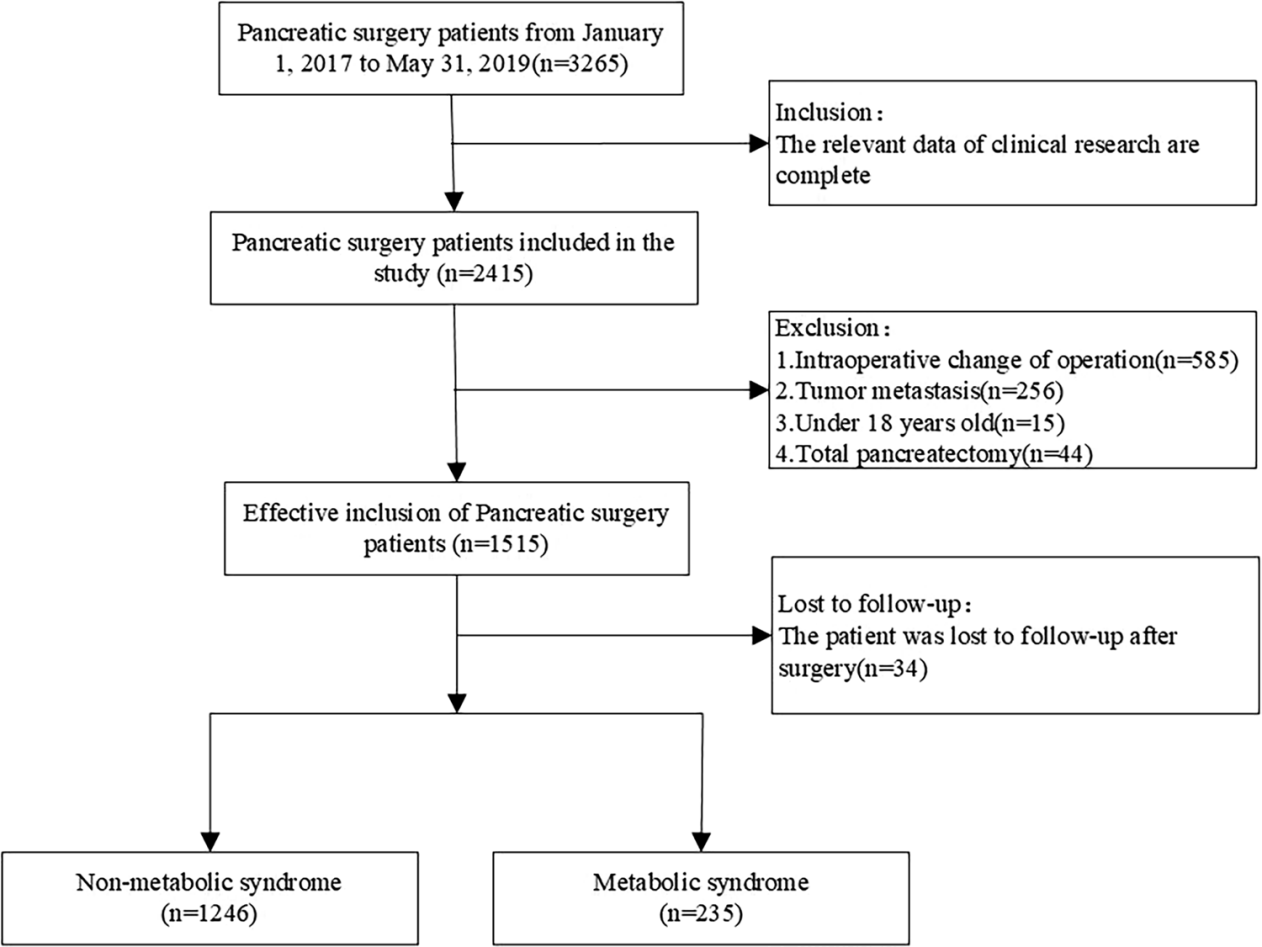
Flowchart for the process of inclusion.

After screening, there were 3265 patients who had pancreatic cancer during the studied period, of whom, 2415 patients met the inclusion criteria and had complete clinical information. After excluding 585 patients who did not receive pancreatic surgery, 256 patients who had tumor metastasis, 15 patients whose were aged under 18, 44 patients underwent total pancreatectomy, and 34 patients were lost of follow-ups, a total of 235 MS patients and 1246 non-MS patients were analyzed in this study (Fig. 1). After the PSM, there were a total of 1163 patients finally left in the study cohort (MS group = 235, control group = 928).

Patients’ characteristics were summarized in Table 1. After PSM, in addition to the diagnostic inclusion of metabolic syndrome, age and ASA were statistically different between the two groups.

**Table 1:** Descriptive data and outcomes of patients.

Parameters	Before PSM			After PSM		
	MS (n = 235)	Non-MS (n = 1246)	*P*	MS (n = 235)	Non-MS (n = 928)	*P*
Gender, n (male%)	155 (66.0)	706 (56.7)	0.008*	155 (66.0)	626 (66.6)	0.853
Age, y	62.8 ± 10.0	59.9 ± 12.5	0.001*	62.8 ± 10.0	60.9 ± 11.7	0.023*
ASA			< 0.001*			< 0.001*
I, n (%)	0 (0.0)	434 (34.8)		0 (0.0)	304 (32.3)	
II, n (%)	91 (38.7)	519 (41.7)		91 (38.7)	408 (43.4)	
III, n (%)	134 (57.0)	293 (23.5)		134 (57.0)	228 (24.3)	
IV, n (%)	10 (4.3)	0 (0.0)		10 (4.3)	0 (0.0)	
Operation time, h	3.0 ± 0.9	2.8 ± 1.0	0.003*	3.0 ± 0.9	2.9 ± 1.0	0.132
Chemotherapy, n (%)	6 (2.6)	32 (2.6)	0.989	6 (2.6)	20 (2.1)	0.692
Biliary stented, n (%)	21 (8.9)	116 (9.3)	0.856	21 (8.9)	84 (8.9)	1.000
Blood transfusion, n (%)	48 (20.4)	264 (21.2)	0.793	48 (20.4)	213 (22.7)	0.461
Operation method			0.006*			0.854
PD, n (%)	156 (66.4)	707 (56.7)		156 (66.4)	618 (65.7)	
PP, n (%)	79 (33.6)	539 (43.3)		79 (33.6)	322 (34.3)	
BMI, kg/m^2^	25.6 ± 2.7	22.4 ± 2.9	< 0.001*	25.6 ± 2.7	22.3 ± 2.9	< 0.001*
Diagnosis of MS						
Hypertension, n (%)	182 (77.4)	237 (19.0)	< 0.001*	182 (77.4)	181 (19.3)	< 0.001*
Hyperlipidemia, n (%)	198 (84.3)	394 (31.6)	< 0.001*	198 (84.3)	323 (34.4)	< 0.001*
Obesity, n (%)	157 (66.8)	202 (16.2)	< 0.001*	157 (66.8)	146 (15.5)	< 0.001*
Diabetes, n (%)	197 (83.8)	426 (34.2)	< 0.001*	197 (83.8)	337 (35.9)	< 0.001*
Total length of stay, d	14 (10.21)	13 (10.18)	0.001*	14 (10.21)	13 (10.19)	0.083
Clavien-Dindo grades			0.052			0.274
0, n (%)	171 (72.7)	971 (77.9)		171 (72.7)	692 (74.6)	
I, n (%)	29 (12.3)	119 (9.5)		29 (12.3)	104 (11.2)	
II, n (%)	31 (13.2)	122 (9.8)		31 (13.2)	105 (11.3)	
III, n (%)	2 (0.9)	20 (1.6)		2 (0.9)	15 (1.6)	
IV, n (%)	0 (0.0)	12 (1.0)		0 (0.0)	10 (1.1)	
V, n (%)	2 (0.9)	2 (0.2)		2 (0.9)	2 (0.2)	
ICU treatment days, d	2 (0.4)	2 (0.3)	0.056	2 (0.4)	2 (0.3)	0.150
Outcomes in 30 days						1.000
Death, n (%)	2 (0.9)	8 (0.6)		2 (0.9)	8 (0.9)	
Survival, n (%)	233 (99.1)	1238 (99.4)		233 (99.1)	932 (99.1)	
Outcomes in 90 days			0.210			0.341
Death, n (%)	5 (2.1)	14 (1.1)		5 (2.1)	14 (1.5)	
Survival, n (%)	230 (97.9)	1232 (98.9)		230 (97.9)	926 (98.5)	

*P value was less than 0.05, with statistical significance.

*MS* Metabolic syndrome, *PSM* Propensity score matching, *Non-MS* Non-metabolic syndrome, *Chemotherapy* Preoperative chemotherapy, *Biliary stented* Preoperative biliary stented, *PD* Pancreatoduodenectomy, *PP* Partial pancreatectomy.

After PSM, intravenous infusion rate, and the volume of perioperative bleeding were significantly different between the two groups (*P* < 0.001, Table 2).

**Table 2:** Intraoperative fluid management between MS and non-MS.

Parameters	Before PSM			After PSM		
	MS (n = 235)	Non-MS (n = 1246)	*P*	MS (n = 235)	Non-MS (n = 940)	*P*
R colloidal/crystal	0.6 (0.3, 0.9)	0.5 (0.3, 0.9)	0.035*	0.6 (0.3, 0.9)	0.5 (0.3, 0.9)	0.034*
Total fluid, L	2.6 (2.1, 3.1)	2.3 (2.1, 3.0)	0.002*	2.6 (2.1, 3.1)	2.6 (2.1, 3.1)	0.068
IV rate, ml/kg/h	12.4 (9.3, 16.3)	14.7 (11.4, 18.9)	< 0.001*	12.4 (9.3, 16.3)	14.2 (10.9, 18.1)	< 0.001*
Bleeding, L	0.4 (0.3, 0.6)	0.3 (0.2, 0.5)	< 0.001*	0.4 (0.3, 0.6)	0.3 (0.2, 0.5)	< 0.001*

*P value was less than 0.05, with statistical significance.

*MS* Metabolic syndrome, *PSM* Propensity score matching, *Non-MS* Non-metabolic syndrome, *R colloidal/crystal* Ratio of colloidal fluid/crystal fluid, *Total fluid* Total volume of fluid, *IV rate* Intravenous fluid rate, *Bleeding* The volume of perioperative bleeding.

### Composite complications

The ORs of MS on postoperative complications after pancreatic surgery were illustrated in Table 3. After PSM, the occurrence of postoperative AKI was significantly different between the two groups (OR: 1.730, 95%CI: 1.050–2.849, *P* = 0.031), and difference in the postoperative composite complications between patients with and those without MS were not different (OR: 1.116, 95%CI: 0.808–1.542, *P* = 0.504). No other postoperative component was found to be significantly different in patients with or without MS (*P* > 0.05).

**Table 3:** Components of Composite complications after open pancreatic surgery.

	Before PSM					After PSM				
	MS (n = 235)	Non-MS(n = 1246)	*P*	OR	95% CI	MS (n = 235)	Non-MS (n = 940)	*P*	OR	95% CI
CC	64 (27.2)	275 (22.1)	0.085	1.322	0.963–1.814	64 (27.2)	236 (25.1)	0.504	1.116	0.808–1.542
AKI	24 (10.2)	65 (5.2)	0.004*	2.067	1.265–3.375	24 (10.2)	58 (6.2)	0.031*	1.730	1.050–2.849
CCE	3 (1.3)	17 (1.4)	0.915	0.935	0.272–3.216	3 (1.3)	15 (1.6)	0.722	0.797	0.229–2.777
PC	10 (4.3)	45 (3.6)	0.633	1.186	0.589–2.388	10 (4.3)	35 (3.7)	0.704	1.149	0.561–2.356
NPPI	43 (18.3)	206 (16.5)	0.507	1.131	0.787–1.625	43 (18.3)	182 (19.1)	0.711	0.933	0.645–1.348
CRSI	3 (1.3)	26 (2.1)	0.416	0.607	0.182–2.021	3 (1.3)	19 (2.0)	0.455	0.627	0.184–2.136

*P value was less than 0.05, with statistical significance.

*MS* Metabolic syndrome, *PSM* Propensity score matching, *Non-MS* Non-metabolic syndrome, *CC* Composite complications, *AKI* Acute kidney injury, *CCE* Cardiovascular and cerebrovascular event, *PC* Pulmonary complications, *NPPI* Non-pulmonary postoperative infection, *CRSI* Complications requiring surgical intervention.

The association between each covariate and MS with the postoperative composite complications were summarized in Table 4. After PSM, the results showed patients who underwent partial pancreatectomy had a lower risk of postoperative CC compared to those who underwent pancreatoduodenectomy (OR: 0.524, 95%CI: 0.366–0.752, *P* < 0.001). A younger age (OR: 0.980, 95%CI: 0.968–0.993, *P* = 0.002) and a shorter operation time (OR: 0.792, 95%CI: 0.689–0.911, *P* = 0.001) were also factors that reduce postoperative CC.

**Table 4:** Covariates and MS on postoperative composite complications after pancreatic surgery.

Parameter	Before PSM			After PSM		
	*P*	OR	95% CI	*P*	OR	95% CI
Operation (PP vs. PD)	< 0.001*	0.521	0.380–0.714	< 0.001*	0.524	0.366–0.752
Age (Young vs. Old)	0.004*	0.984	0.973–0.995	0.002*	0.980	0.968–0.993
Gender (F vs. M)	0.034*	0.770	0.604–0.981	0.275	0.864	0.665–1.123
Low IV rate	0.771	0.997	0.974–1.019	0.400	0.990	0.967–1.014
Short operation time	< 0.001*	0.775	0.686–0.876	0.001*	0.792	0.689–0.911
MS (Non-MS vs. MS)	0.519	0.907	0.676–1.219	0.771	0.958	0.715–1.282

*P value was less than 0.05, with statistical significance. MS: Metabolic syndrome. Operation (PP vs. PD): Operation of partial pancreatectomy versus operation of pancreatoduodenectomy; Gender (F vs. M): Female versus male in term of gender; Age (Young vs. Old): Young age versus old age; Low IV rate: low intravenous infusion rate; Short operation time: short duration of operation time.

### Survival analysis

**Figure 2:**
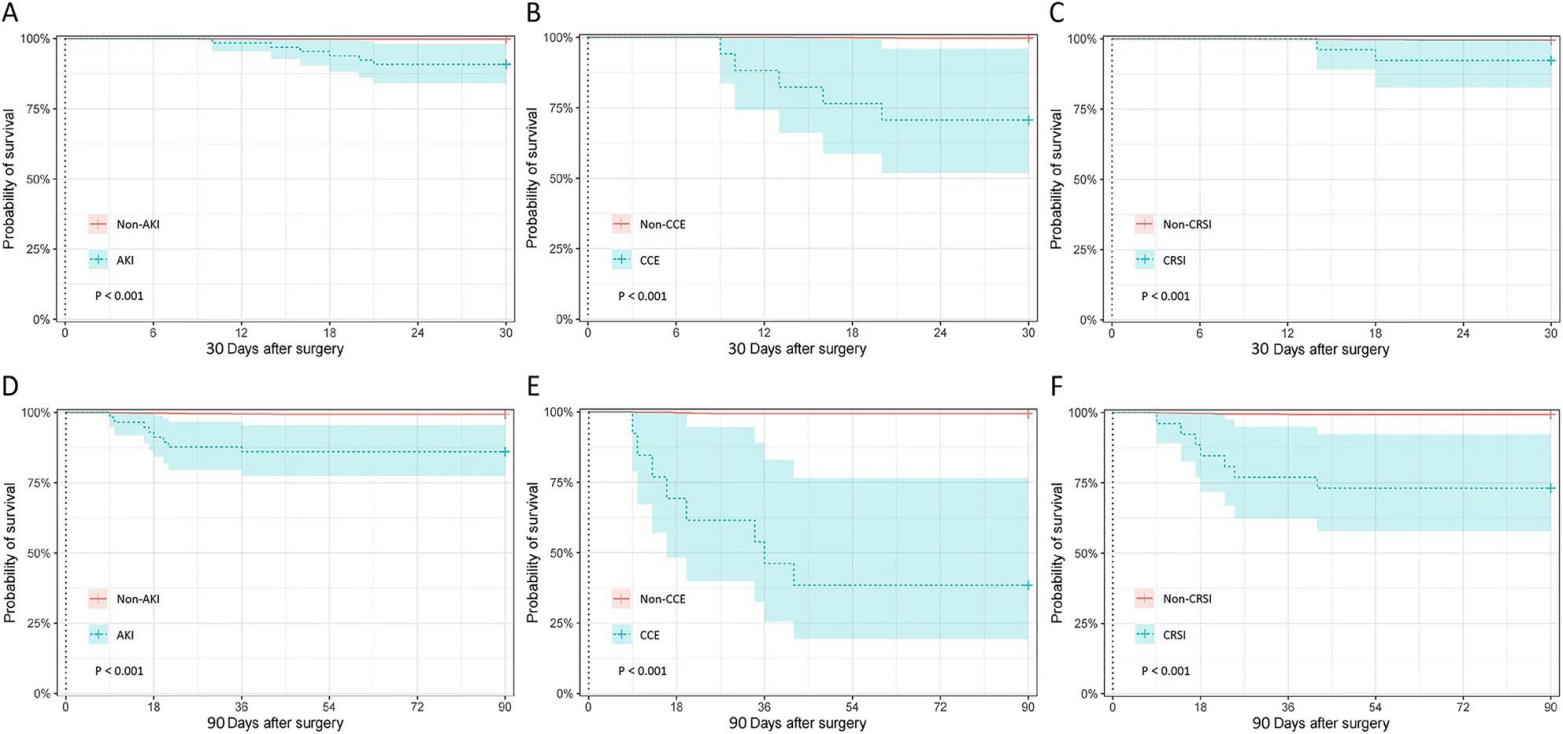
Cox survival curves of CCE, AKI and CSRI. CCE: cardiovascular and cerebrovascular event; AKI: Acute Kidney Injury; CRSI: complications requiring surgical intervention.

CCE, AKI, and CSRI were significantly associated with both 30-day and 90-day mortality in multivariate cox regression, whereas PC was only significantly associated with 90-day mortality (All *P* < 0.001). The detailed data of multivariate COX regression were shown in Table 5. Cox survival curves of CCE, AKI and CSRI were illustrated in Fig. 2.

**Table 5:** Multivariate Cox regression analysis of patients within 30 days or 90 days after surgery.

Parameter	30 days after surgery			90 days after surgery		
	*P*	OR	95% CI	*P*	OR	95% CI
CCE	0.000*	28.915	5.305–157.598	0.000*	43.986	15.079–128.307
AKI	0.001*	20.196	3.653–111.638	0.000*	11.478	3.754–35.098
CRSI	0.001*	15.959	3.215–79.217	0.000*	34.375	11.694–101.040
PC	0.281	2.212	0.522–9.370	0.001*	5.754	1.955–16.940
NPPI	0.204	3.218	0.530–19.555	0.920	1.058	0.351–3.191
MS	0.804	1.229	0.241–6.261	0.725	0.761	0.166–3.487

*P value was less than 0.05, with statistical significance.

*CCE* Cardiovascular and cerebrovascular event, *AKI* Acute kidney injury, *CRSI* Complications requiring surgical intervention, *PC* Pulmonary complications, *NPPI* Non-pulmonary postoperative infection, *MS* Metabolic syndrome.

**Figure 3:**
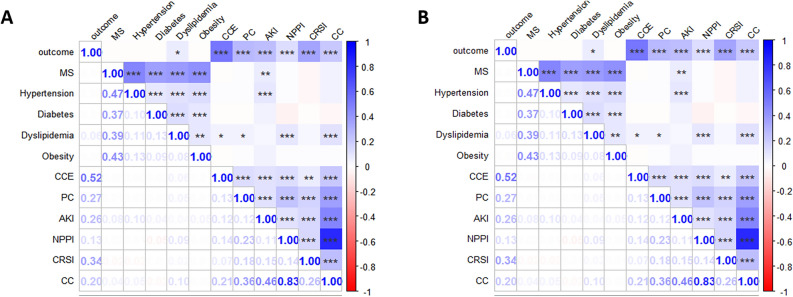
Overall correlations between outcomes and MS or CC and their components. ***P < 0.001, **P < 0.01,*P < 0.05. (**A**) 30-day spearman results; (**B**) 90-day spearman results. MS: Metabolic syndrome; HT: hypertension; DM: diabetes mellitus; DL: dyslipidemia; OO: overweight or obesity; CCE: cardiovascular and cerebrovascular event; PC: pulmonary complications; AKI: Acute Kidney Injury; NPPI: non-pulmonary postoperative infection; CRSI: complications requiring surgical intervention; CC: composite complications.

Overall correlations between the outcomes within 30 and 90 days after the surgery and MS, CC and their components were illustrated in Fig. 3.

## Discussion

Although the diagnostic criteria for MS adopted differently, the percentage of MS patients in pancreatic cancer (15.9%) was consistent with the previous studies (11–24%)^18,23^.

Postoperative complications of pancreatic surgeries were complex^9,24^. After PSM, GEE was employed to analyze the overall complications without discussing the distribution form of dependent variables, which fits a marginal model in the context of longitudinal studies^25,26^. There were no significant association between MS and postoperative composite complications after open pancreatic surgery (OR = 0.958, 95%CI: 0.715–1.282, *P* = 0.771). It indicated that MS did not increase the overall risk in postoperative complications after pancreatic surgery independently.

The conclusion of our analysis was consistent with some studies^18,27^. However, May C Tee et al. reported that MS patients who received selective pancreatic surgery had an increased risk of postoperative morbidity and some complications^9^. It inferred that MS may increase some certain complications.

In our results, AKI was significantly associated with MS after PSM (OR: 1.730, 95%CI: 1.050–2.849, *P* = 0.031). Several studies on cardiac surgery have indicated that MS patients were associated with increased rates of postoperative morbidity, infections, cardiovascular and renal adverse events^14–16^. Congruently, our research demonstrated that MS patients was susceptible to postoperative AKI after pancreatic surgery.

MS components such as obesity, hypertension, elevated TG, low HDL-C, impaired fasting glucose, and MS per se were contributed to decreased GFR^28^. The Chinese Diabetes Society (CDS) diagnostic criteria of MS adopted a relatively smaller BMI standard (BMI ≥ 25) for Chinese population compared to the western population. The smaller BMI did not prevent occurrence of AKI, although there is a possibility that the risk of postoperative AKI after non-cardiac surgery would be increased with severity of obesity^29^.

Intraoperative fluid therapy may affect postoperative AKI. Restrict fluid therapy was not used in our center for the pancreatic surgery (in Table 2). According to the previous studies that restricted infusion is more likely to cause postoperative AKI, the statistical differences in intraoperative fluid therapy may not be the key point for AKI^30^. Multivariate regression analysis showed that intraoperative bleeding was an independent risk factor for postoperative AKI compared with intraoperative volume management (in Supplementary table). It was suggested that strategies to reduce intraoperative bleeding may help to reduce AKI after pancreatic surgery.

Although NPPI accounted for 80% of CC and all complications were associated with outcomes in the spearman analysis, only CCE, AKI and CRSI were associated with survival within 30 and 90 days of surgery after multivariate Cox proportional hazards model analysis. It was implicated that the effective prevention of CCE, AKI and CRSI after pancreatic surgery is helpful to reduce postoperative mortality rate. They require more attention from anesthesiologists and surgeons due to their impact on 30-day and 90-day mortality following pancreatic surgery. Because AKI is more susceptible and increases perioperative mortality for MS patients after the pancreatic surgery. It indicated that MS patients received pancreatic surgery should be paid more attention to postoperative AKI for both anesthesiologists and surgeons.

There are several limits in this study. Firstly, this is a single-centered retrospective study, which largely limited the quality of prognosis. But fortunately, our center is a high volume of pancreatic center, and patients received from all over the country. Because the study is based on Chinese diagnostic criteria, patients across the country can provide a certain degree of reference. Secondly, the definition of MS adopted in this study relied on the CDS criteria published in 2004 exclusively characterized for Chinese population^31^, which might inherently differentiate the characteristics of our patients from those in previous studies. Yet the influence of different diagnostic criteria for MS on postoperative complications is our study.

In conclusion, we observed that MS patients who received open pancreatic surgery had no association with postoperative composite complications during hospitalization. But MS is an independent risk factor for postoperative AKI of pancreatic surgery in Chinese population. And AKI was associated with perioperative survival of MS.

## Supplementary Information

Supplementary Information 1.

Supplementary Information 2.

Supplementary Information 3.

## Data Availability

The datasets generated and analyzed during the current study are available from Dryad Digital Repository (https://doi.org/10.5061/dryad.bcc2fqzdz) and can be temporarily visited by https://datadryad.org/stash/share/OEWZ_1qNX9ijRMg86tHeNUUJiOIBLiOkoRc4sCGcKQs.

## References

[1] Siegel RL, Miller KD, Fuchs HE, Jemal A (2021). Cancer statistics, 2021. CA Cancer J. Clin..

[2] GBD 2017 Pancreatic Cancer Collaborators (2017). The global, regional, and national burden of pancreatic cancer and its attributable risk factors in 195 countries and territories, 1990–2017: A systematic analysis for the Global Burden of Disease Study 2017. Lancet Gastroenterol. Hepatol..

[3] Ma J, Siegel R, Jemal A (2013). Pancreatic cancer death rates by race among US men and women, 1970–2009. J. Natl. Cancer Inst..

[4] Mizrahi JD, Surana R, Valle JW, Shroff RT (2020). Pancreatic cancer. Lancet.

[5] Okasha H (2017). Real time endoscopic ultrasound elastography and strain ratio in the diagnosis of solid pancreatic lesions. World J. Gastroenterol..

[6] Vollmer CM (2012). A root-cause analysis of mortality following major pancreatectomy. J. Gastrointest. Surg..

[7] Kelly KJ (2011). Risk stratification for distal pancreatectomy utilizing ACS-NSQIP: Preoperative factors predict morbidity and mortality. J. Gastrointest. Surg..

[8] Rosato V (2011). Metabolic syndrome and pancreatic cancer risk: A case-control study in Italy and meta-analysis. Metabolism.

[9] Tee MC (2016). Metabolic syndrome is associated with increased postoperative morbidity and hospital resource utilization in patients undergoing elective pancreatectomy. J Gastrointest Surg.

[10] Chang EH (2020). Obesity and surgical complications of pancreaticoduodenectomy: An observation study utilizing ACS NSQIP. Am. J. Surg..

[11] Tan DJH (2021). The influence of diabetes on postoperative complications following colorectal surgery. Tech. Coloproctol.

[12] Gu A (2020). Postoperative complications and impact of diabetes mellitus severity on revision total knee arthroplasty. J. Knee Surg..

[13] Sánchez-Guillén L (2019). Risk factors for leak, complications and mortality after ileocolic anastomosis: Comparison of two anastomotic techniques. Ann. R. Coll. Surg. Engl..

[14] Echahidi N (2007). Metabolic syndrome increases operative mortality in patients undergoing coronary artery bypass grafting surgery. J. Am. Coll. Cardiol..

[15] Tzimas P (2015). Impact of metabolic syndrome in surgical patients: Should we bother?. Br. J. Anaesth..

[16] Hong S, Youn YN, Yoo KJ (2010). Metabolic syndrome as a risk factor for postoperative kidney injury after off-pump coronary artery bypass surgery. Circ. J..

[17] Zhou H (2010). Evidence on the applicability of the ATPIII, IDF and CDS metabolic syndrome diagnostic criteria to identify CVD and T2DM in the Chinese population from a 6.3-year cohort study in mid-eastern China. Diabetes Res. Clin. Pract..

[18] Le Bian AZ (2017). Consequences of metabolic syndrome on postoperative outcomes after pancreaticoduodenectomy. World J. Gastroenterol..

[19] Raviv NV, Sakhuja S, Schlachter M, Akinyemiju T (2017). Metabolic syndrome and in-hospital outcomes among pancreatic cancer patients. Diabetes Metab. Syndr.

[20] Téoule P (2020). Obesity and pancreatic cancer: A matched-pair survival analysis. J. Clin. Med..

[21] Lee SH, Tao S, Kim HS (2019). The prevalence of metabolic syndrome and its related risk complications among Koreans. Nutrients.

[22] Savadatti SS (2019). Metabolic syndrome among Asian Indians in the United States. J. Public Health Manag. Pract..

[23] Xia B (2020). Metabolic syndrome and risk of pancreatic cancer: A population-based prospective cohort study. Int. J. Cancer.

[24] Karim SAM, Abdulla KS, Abdulkarim QH, Rahim FH (2018). The outcomes and complications of pancreaticoduodenectomy (Whipple procedure): Cross sectional study. Int. J. Surg.

[25] Liu J, Colditz GA (2018). Relative efficiency of unequal versus equal cluster sizes in cluster randomized trials using generalized estimating equation models. Biom. J..

[26] Turan A (2020). Preoperative vitamin D concentration and cardiac, renal, and infectious morbidity after noncardiac surgery. Anesthesiology.

[27] Bhayani NH (2012). Effect of metabolic syndrome on perioperative outcomes after liver surgery: A National Surgical Quality Improvement Program (NSQIP) analysis. Surgery.

[28] Yu M, Ryu DR, Kim SJ, Choi KB, Kang DH (2010). Clinical implication of metabolic syndrome on chronic kidney disease depends on gender and menopausal status: Results from the Korean National Health and Nutrition Examination Survey. Nephrol. Dial. Transplant..

[29] Glance LG (2010). Perioperative outcomes among patients with the modified metabolic syndrome who are undergoing noncardiac surgery. Anesthesiology.

[30] Myles PS (2018). Restrictive versus liberal fluid therapy for major abdominal surgery. N. Engl. J. Med..

[31] Li Y, Zhao L, Yu D, Wang Z, Ding G (2018). Metabolic syndrome prevalence and its risk factors among adults in China: A nationally representative cross-sectional study. PLoS ONE.

