# Supplementary material for: Metabolic syndrome for the prognosis of postoperative complications after open pancreatic surgery in Chinese adult: a propensity score matching study
Source: Sci Rep. 2023 Mar 8;13:3889. doi: 10.1038/s41598-023-31112-x (PMC9995346; doi:10.1038/s41598-023-31112-x)
Supplement: Supplementary file 1 — Supplementary Information 1. [file 41598_2023_31112_MOESM1_ESM.docx]

**Supplementary figure legend**

Supplementary Figure. Absolute standardized difference in means of concerned data before and after two different propensity score matching. A: propensity matching with gender, operation method, preoperative biliary stented and preoperative chemotherapy; B: propensity matching with gender, operation method, and preoperative biliary stented
