# Supplementary figures and images for: Metabolic syndrome for the prognosis of postoperative complications after open pancreatic surgery in Chinese adult: a propensity score matching study
Source: Sci Rep. 2023 Mar 8;13:3889. doi: 10.1038/s41598-023-31112-x (PMC9995346; doi:10.1038/s41598-023-31112-x)

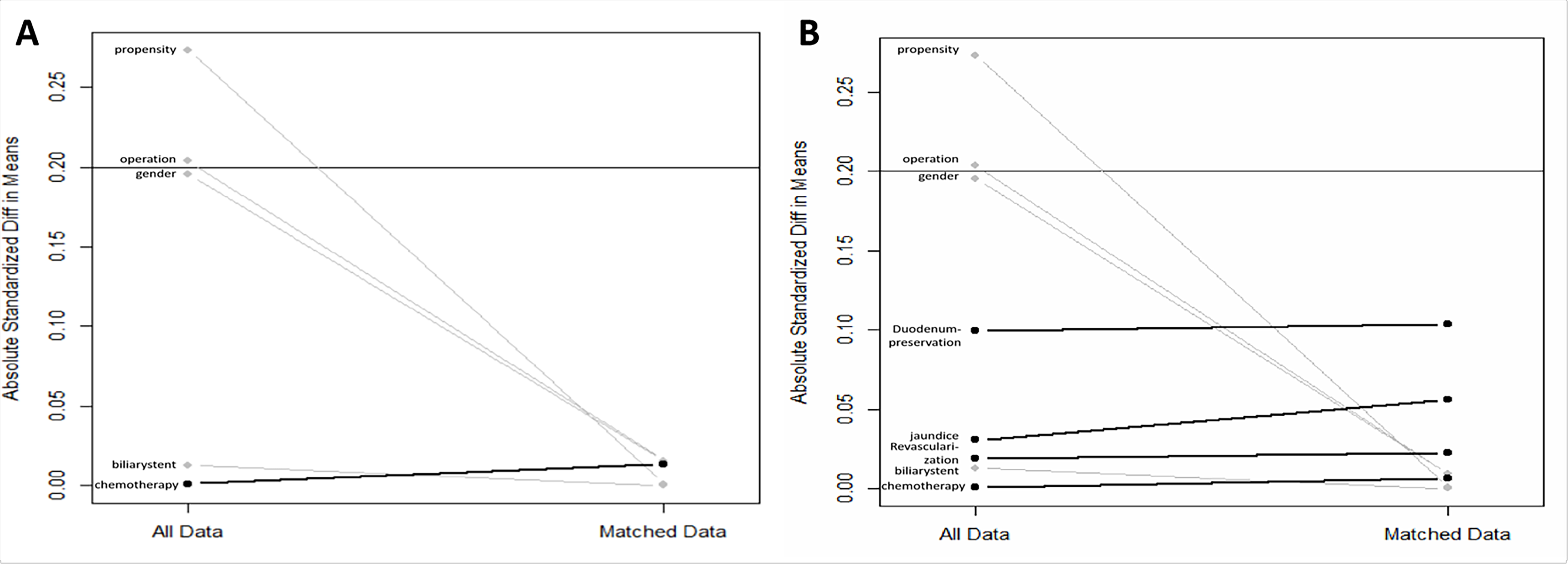

Supplement: Supplementary file 2 — Supplementary Information 2. [file 41598_2023_31112_MOESM2_ESM.tiff]
