# Supplementary material for: Metabolic syndrome for the prognosis of postoperative complications after open pancreatic surgery in Chinese adult: a propensity score matching study
Source: Sci Rep. 2023 Mar 8;13:3889. doi: 10.1038/s41598-023-31112-x (PMC9995346; doi:10.1038/s41598-023-31112-x)
Supplement: Supplementary file 3 — Supplementary Information 3. [file 41598_2023_31112_MOESM3_ESM.docx]

**Supplement Table** Multivariate logistic analysis of postoperative AKI

| Parameters | before PSM | | | After PSM | | |
| --- | --- | --- | --- | --- | --- | --- |
|  | P | OR | 95% CI | P | OR | 95% CI |
| crystal fluid | 0.062 | 2.267 | 0.958-5.361 | 0.365 | 1.550 | 0.601-3.996 |
| colloidal fluid | 0.859 | 1.086 | 0.439-2.688 | 0.888 | 0.933 | 0.354-2.458 |
| Ratio of colloidal fluid/crystal fluid | 0.223 | 1.490 | 0.784-2.833 | 0.360 | 1.375 | 0.695-2.721 |
| Fluid infusion rate | 0.007^*^ | 0.938 | 0.896-0.982 | 0.194 | 0.969 | 0.925-1.016 |
| Intraoperative blood transfusion | 0.656 | 0.812 | 0.325-2.028 | 0.732 | 0.846 | 0.324-2.204 |
| Total liquid infusion volume | 0.685 | 0.857 | 0.406-1.807 | 0.759 | 0.885 | 0.406-1.928 |
| Bleeding | 0.002^*^ | 2.035 | 1.288-3.215 | 0.002^*^ | 2.060 | 1.293-3.283 |
| Urine volume | 0.214 | 0.615 | 0.285-1.324 | 0.470 | 0.746 | 0.336-1.653 |

*P value was less than 0.05, with statistical significance. AKI: Acute Kidney Injury.
